# The effect of varying water volumes on in vivo dissolution and gastric emptying of highly soluble caffeine and lesser soluble theobromine containing capsules

**DOI:** 10.1016/j.ijpx.2026.100615

**Published:** 2026-07-15

**Authors:** Linus Großmann, Johanna Cyrus, Stefan Senekowitsch, Toni Wildgrube, Marie-Luise Kromrey, Werner Weitschies, Philipp Schick, Michael Grimm

**Affiliations:** a University of Greifswald, Institute of Pharmacy, Dep. of Biopharmaceutics and Pharmaceutical Technology, Felix-Hausdorff-Str. 3, 17489 Greifswald, Germany; b Martin-Luther-University Halle-Wittenberg, Institute of Pharmacy, Dep. of Pharmaceutical Technology, 06099 Halle, Germany; c University Medicine Rostock, Institute of Pharmacology and Toxicology, Schillingallee 70, 18057 Rostock, Germany; d University Medicine Carl Gustav Carus, Institute of Diagnostic and Interventional Radiology, Fetscherstraße 74, 01307 Dresden, Germany

**Keywords:** *Magenstrasse*, Gastric Emptying, In Vivo Dissolution, Oral Drug Absorption, Saliva, Caffeine, Theobromine

## Abstract

The influence of co-administered water volume on the intragastric dissolution and the systemic exposure of drugs taken under fed conditions remains clinically relevant, particularly for drug substances with limited aqueous solubility. This secondary pharmacokinetic analysis, based on a previously published MRI study (*n* = 12) evaluated the effect of 50, 100, and 150 mL water on the salivary pharmacokinetics of ^13^C₃-caffeine (25 mg; high solubility, high permeability) and theobromine (50 mg; lower solubility, high permeability) administered in a hard gelatin capsule 30 min after a standardized meal. Saliva samples were collected over 119 min and quantified by LC–MS/MS. Both compounds appeared rapidly in saliva irrespective of the initial water volume, indicating that even 50 mL effectively triggered postprandial liquid emptying via the *Magenstrasse*. Wilcoxon Signed-Rank test of dose-normalized AUC_0__–__119 min_ demonstrated no significant differences between both substances. Phase-specific analysis (0–59 min vs. 59–119 min) revealed a strong *phase* effect (*p* < 0.001) and a significant *phase × substance* interaction (p < 0.001), but no effect of initial *fluid volume*, reflecting a pronounced late exposure increase for theobromine after administration of an additional 240 mL water at 60 min. These findings indicate that small fluid volumes are sufficient to initiate rapid gastric transit of dissolved fractions under fed conditions. However, for less soluble compounds, undissolved fractions may persist intragastrically and contribute to delayed, biphasic absorption following subsequent fluid intake. Standardization of post-dose drinking behavior should therefore be considered in food-effect and bioavailability studies.

## Graphical abstract

**Figure:**
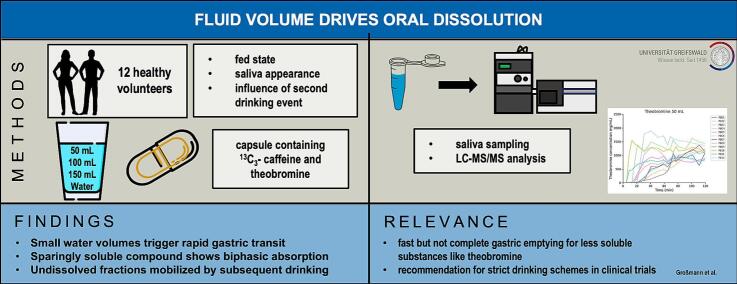

## 1 Introduction

In a previous study, we demonstrated that the *Magenstrasse*, an accelerated pathway for water past the chyme in the postprandial stomach, operates independently of the volume of water administered (Großmann et al., 2025). Even when administering volumes of water smaller than the 240 mL recommended by FDA (Food & Drug Administration) guidelines for bioequivalence and food effect studies, the stomach is emptied from the ingested water after just 20–30 min (European Medines Agency, 2024). In addition, it was demonstrated that, regardless of the administered volume, the caffeine released from immediate-release dosage forms is not entirely eliminated by the initial water emptying and is therefore not fully available for absorption in the small intestine. This was attributed to various factors, including the different densities of capsules (floating) and tablets (sinking) and the resulting location in the stomach, the hydrophilic test meal and the time required for the dosage form to liberate the active ingredient and dissolution. Drinking 240 mL of water one hour later caused the emptying of the remaining caffeine from the stomach, causing a rather small secondary increase in the measured caffeine saliva levels.

Since not all active pharmaceutical ingredients (APIs) correspond to BCS class I (Biopharmaceutic Classification System) and are therefore highly soluble and permeable, such as caffeine, the question arises as to how APIs with different physicochemical properties, such as poorer solubility, react to varying volumes of co-administered water (Smetanova et al., 2009; Samineni et al., 2022).

The amount of water ingested with oral drug formulations critically affects gastrointestinal transit, luminal drug dissolution, and systemic absorption. While regulatory guidelines recommend administration with 150–240 mL water under standardized fasted or fed state conditions (FDA and CDER, 2003; EMA and CDER, 2010; FDA and CDER, 2002), observational studies indicate that many patients take their medication with substantially smaller volumes, often less than 100 mL (Hens et al., 2017; Sarwinska et al., 2024). Such discrepancies may contribute to variability in pharmacokinetics in general and bioavailability in particular, especially for compounds whose absorption is limited by solubility or permeability.

The BCS provides a mechanistic framework for predicting the sensitivity of drug absorption to intraluminal conditions. BCS Class I compounds, characterized by high solubility and high permeability, are generally robust to variations in fluid volume (EMA and CDER, 2010; EMA et al., 2020; Tsume et al., 2014). In contrast, Class II compounds (low solubility, high permeability) depend heavily on sufficient luminal volume to facilitate dissolution and prevent precipitation, especially in the upper gastrointestinal tract (Nader et al., 2016; Van Den Abeele et al., 2016). Recent in vivo studies, partly performed in rats, confirm that small fluid volumes delay dissolution and reduce C_max_ or increase interindividual variability for BCS II drugs, even when total exposure (AUC, area under the curve) remains unaffected (Kataoka et al., 2022; Vinarov et al., 2021; Danielak et al., 2024). Under fed conditions, these effects are further modulated by altered gastric emptying dynamics and solubilizing effects of food components, making BCS II drugs particularly sensitive to the interplay between formulation, fluid volume, and gastrointestinal state (Rodriguez-Fernandez et al., 2025; Kambayashi and Shirasaka, 2023; Tzakri et al., 2023; Romański et al., 2024).

In this context, we employed theobromine as a model compound, as it also appears in the saliva like caffeine, correlates well with plasma concentrations, but exploits an overall lower solubility (Rodopoulos et al., 1996). In comparison to caffeine, it combines low aqueous solubility (∼ 0.33 mg/mL versus ∼21.7 mg/mL at 25 °C) with high gastrointestinal permeability and systemic exposure in both animals and humans (Baggott et al., 2013; Gołdyn et al., 2019; Guillory, 2003). Its structural similarity to methylxanthines such as caffeine and theophylline facilitates resembling analytical tracking, while its pharmacokinetic behavior renders it likely suitable for probing dissolution-limited absorption under controlled conditions (Tzakri et al., 2023; Ptolemy et al., 2010; Sager et al., 2018). Theobromine thus could serve as a biopharmaceutically relevant surrogate to investigate the impact of co-administered fluid volume on the in vivo performance of sparingly soluble, highly permeable compounds, particularly given that its complete intragastric dissolution is critically dependent on the available fluid volume and contact time, both of which are limited under postprandial conditions due to the rapid gastric water emptying via the Magenstrasse.

This analysis extends a prior randomized crossover study involving healthy participants who received a hard gelatin capsule containing 25 mg ^13^C₃-caffeine and 50 mg theobromine, administered with 50, 100, or 150 mL of water under standardized postprandial conditions. Saliva samples were collected over a 120 min period and analyzed using liquid chromatography coupled with tandem mass spectrometry (LC-MS/MS), and gastric water emptying was assessed via MRI (Magnetic Resonance Imaging). Caffeine and theobromine were chosen as model compounds based on their distinct aqueous solubilities. The objective was to examine how these physicochemical differences influence intraluminal dissolution, gastric emptying and systemic exposure across varying hydration levels and *Magenstrasse* conditions.

## 2 Material & methods

### 2.1 Study design

This secondary pharmacokinetic analysis is based on a previously published randomized, three-arm, crossover MRI study conducted in 12 healthy subjects (5 female, 7 male, 26.1 ± 5.1 years, 74.3 ± 11.8 kg, 179.9 ± 7.0 cm, 22.9 ± 3.0 kg/m^2^) under standardized postprandial conditions (Großmann et al., 2025). The MRI measurements, including gastric content volumetry and intragastric localization of the dosage forms, were performed as part of the original study protocol and are described and reported in full in (Großmann et al., 2025); they are not repeated here to avoid redundancy. For the purposes of the original study, theobromine was included in the capsule formulation as an additional model compound but was not reported, as it fell outside the scope of that publication. The present manuscript constitutes the first dedicated report of these theobromine data. Briefly, each subject received a standardized light meal followed by administration of a gelatin hard capsule with either 50, 100, or 150 mL of water, and saliva samples were collected over 119 min following administration. The study was approved by the Ethics Committee of the University Medicine Greifswald (BB 080/23), registered in the German Clinical Trials Register (DRKS00033695) and followed the rules of the Declaration of Helsinki in its most recent form. All subjects provided written informed consent.

### 2.2 Dosage form and model substances

The size 0 gelatin hard capsule (WEPA Apothekenbedarf GmbH & Co KG, Germany) contained 25 mg of stable isotope-labelled ^13^C₃-caffeine (Sigma-Aldrich Chemie GmbH, Germany) and 50 mg of theobromine (Alfa Aesar Chemicals, Germany). Additionally, a smaller size 3 gelatin hard capsule (WEPA Apothekenbedarf GmbH & Co KG, Germany) filled with approximately 240 mg of medium-chain triglycerides (MCT) (Caesar & Loretz GmbH, Germany) was inserted in the size 0 capsule to enable MRI localization based on its fat signal. The resulting capsule had an approximate density of 0.68 g/mL (Großmann et al., 2025).

### 2.3 Study protocol

The study followed a randomized, partially counterbalanced crossover design. All subjects completed three study visits with at least a 5-day washout interval. After an overnight fast (≥10 h) and caffeine and theobromine abstinence (≥72 h), participants consumed a standardized light meal (two toasted wheat bread slices with butter and strawberry jam, strawberry yoghurt and orange juice; 596 kcal) within 15 min. 30 min after the start of the meal, the capsule was administered with either 50 (A), 100 (B), or 150 mL (C) of water, depending on the study arm. The capsule was administered in an upright position together with the respective water volume, after which subjects immediately assumed a supine position inside the MRI scanner for 59 min with regular MR imaging and saliva sampling. At *t* = 60 min, an additional 240 mL of water was administered in the upright position, after which subjects remained upright. After this, saliva sampling only continued until *t* = 119 min (Großmann et al., 2025).

### 2.4 Saliva sampling and LC-MS/MS

Saliva samples were collected via unstimulated drooling into 2 mL Eppendorf tubes at 19 predefined time points: *t* = −5, 5, 9, 13, 17, 21, 31, 41, 51, 59, 65, 69, 73, 77, 81, 91, 101, 111, and 119 min and stored at −80 °C until analysis. Sample preparation, chromatographic conditions, and full bioanalytical validation details — including calibration range (10–2000 ng/mL), quality control samples, internal standard (d9-caffeine, 4.0 μg/mL), and stability assessments in accordance with EMA ICH M10 guidelines (EMA; ICH, n.d.), are described in detail in our preceding publication (Großmann et al., 2025), as the theobromine data reported here were collected within the same study using the identical analytical platform and sample preparation procedure. Additional bioanalytical validation data and representative LC-MS/MS chromatograms are provided in the Supplementary Material. Briefly, theobromine was quantified on a Shimadzu LCMS-8060 system equipped with a Kinetex PS C18 column (150 × 2.1 mm, 2.6 μm) under isocratic conditions (water with 0.1% formic acid / methanol, 78/22 *v*/v; flow rate 0.4 mL/min; oven temperature 40 °C) using positive MRM mode with a transition of 181.00 → 138.20 *m*/*z*, a collision energy of −19.0 eV, and a retention time of 1.60 min (Großmann et al., 2025; EMA; ICH, n.d.).

### 2.5 Pharmacokinetic evaluation & statistics

Time–concentration profiles were analyzed for each subject and water volume. The area under the concentration–time curve (AUC) was computed by the linear trapezoidal rule using a custom Python script. Partial AUCs were determined for an early phase (0–59 min) and a late phase (59–119 min) to capture the impact of the additional 240 mL of water administered at 60 min. The onset difference (first salivary appearance) between theobromine and caffeine was calculated via subtraction of the first time points above the lower limit of quantification. For statistics, all AUC values were normalized to the administered dose. AUC_0__–__119 min_ were compared using a Wilcoxon Signed-Rank test with Bonferroni-correction.

A linear mixed-effects model (LMM) was employed to account for the repeated-measures crossover design and to simultaneously evaluate the effects of drinking volume, substance, and time phase on dose-normalized AUC. Participant identity was included as a random intercept. In the phase evaluation model, fixed effects comprised *drinking volume* (50, 100, 150 mL), *substance* (theobromine vs. ^13^C₃-caffeine), *phase* (0–59 vs. 59–119 min) and all two- and three-way interactions. Models assumed Gaussian residuals and were fitted by restricted maximum likelihood (REML) with Satterthwaite approximation for degrees of freedom. Model fit was assessed using marginal and conditional R^2^ values. Residual diagnostics confirmed normality (Kolmogorov–Smirnov and Shapiro–Wilk tests, both *p* > 0.3). Post-hoc contrasts were Bonferroni-corrected, and statistical significance was assumed at α = 0.05 for all experiments. All analyses were conducted in jamovi (version 2.6) using the GAMLj module (R version 4.4) (Gallucci, n.d.-a; *R Core Team A Language and Environment for Statistical Computing*, n.d.; *The jamovi project Jamovi*, n.d.; Gallucci, n.d.-b).

## 3 Results

All twelve participants completed the study, and no adverse events were reported. One additional subject had to be recruited due to a technical dropout during MRI measurements in the original trial, resulting in a full dataset for twelve evaluable individuals.

**Fig. 1:**
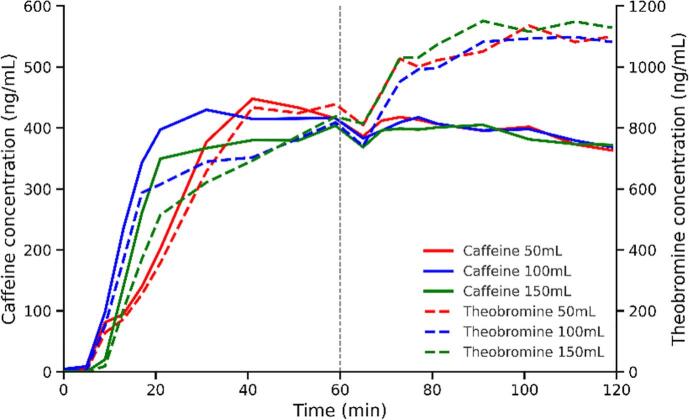
Salivary concentrations of ^13^C_3_-caffeine (25 mg orally, solid lines) and theobromine (50 mg orally, dotted lines) after different co-administered volumes of water (50 mL – red, 100 mL – blue, 150 mL – green) and additional administration of 240 mL water after 60 min (grey dotted line). Mean of *n* = 12, mean. Standard deviations not displayed for clear visibility. (For interpretation of the references to colour in this figure legend, the reader is referred to the web version of this article.)

**Fig. 2:**
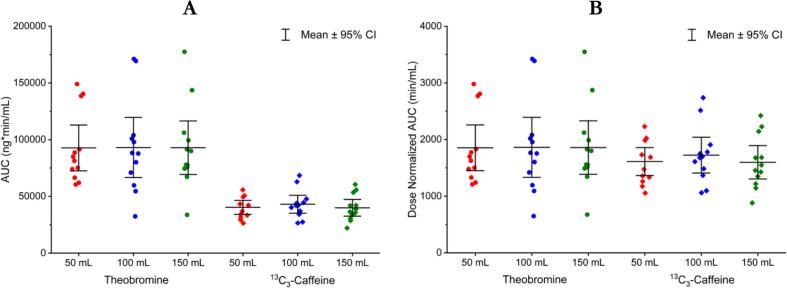
A: Boxplot of AUC_0__–__119 min_ from salivary concentrations of ^13^C_3_-caffeine (diamonds) and theobromine (dots). B: Boxplot of AUC_0__–__119 min_ from dose normalized salivary concentrations of ^13^C_3_-caffeine (diamonds) and theobromine (dots). *n* = 12, individual values, means, 95% confidence intervals (whiskers).

Both caffeine and theobromine showed a similar increase in saliva concentration, regardless of the initial fluid volume administered (Fig. 1). There was a significant difference between the absolute AUCs of ^13^C_3_-caffeine and theobromine (Fig. 2, A). Overall high interindividual variability contributed to high standard deviations in both compounds, with coefficients of variation (CV%) for the dose-normalized AUC_0–119 min_ ranging from 24 to 29% for caffeine and 34–45% for theobromine across all three study arms (spaghetti plots in supplementary files Fig. S1-S6).

Across study arms, the onset of salivary appearance did not differ consistently between theobromine and caffeine. In the 50 mL arm, the mean onset difference (theobromine – caffeine) was −0.3 ± 9.6 min, in the 100 mL arm 1.7 ± 5.3 min, and in the 150 mL arm 0.0 ± 4.6 min. These findings indicate that, on average, both methylxanthines appeared in saliva at nearly the same time, but with considerable interindividual variability in whether caffeine or theobromine was detected first.

After normalization to the given dose, the AUC_0__–__119 min_ did not differ significantly between caffeine and theobromine (p_50 mL_ = 0.330, p_100 mL_ = 1.000, p_150 mL_ = 0.192) using a Wilcoxon Signed-Rank test (Fig. 2, B).

**Fig. 3:**
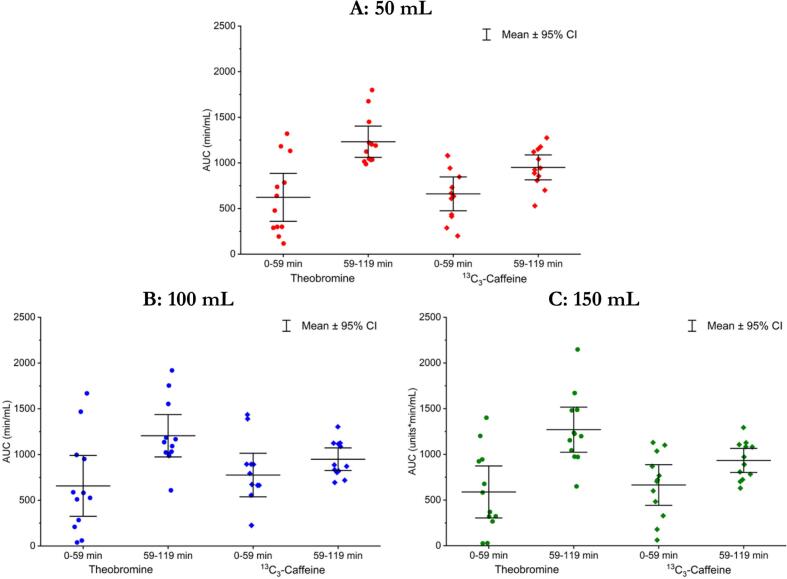
Dose normalized AUC comparison between different phases and salivary tracers theobromine (dots) and ^13^C_3_-caffeine (diamonds). Early phase from 0 to 59 min and late phase from 59 to 119 min. A: 50 mL(red), B: 100 mL (blue), C: 150 mL (green). *n* = 12, individual values, means, 95% confidence intervals (whiskers). (For interpretation of the references to colour in this figure legend, the reader is referred to the web version of this article.)

However, the phase-specific statistical evaluation between an early (0–59 min) and a late phase (59–119 min) of dose-normalized salivary exposure demonstrated clear differences between caffeine and theobromine using a LLM (Fig. 3). A strong effect of *phase* was present, with overall higher exposure in the late phase compared to the early phase (estimate = 429, 95% CI: 342–515, *p* < 0.001). The effect of *substance* was significant (estimate = 107, 95% CI: 21–193, *p* = 0.016), and a pronounced *phase × substance* interaction was detected (estimate = 369, 95% CI: 197–542, *p* < 0.001). This interaction reflected a divergent temporal profile: during the early phase, caffeine and theobromine exhibited comparable levels, whereas in the late phase theobromine showed a substantially greater increase in exposure. Post-hoc contrasts confirmed that theobromine values in the late phase exceeded those of caffeine by nearly 300 units on average (p < 0.001). Neither the main effect of *drinking volume* nor its interactions with *phase* or *substance* were significant (all *p* > 0.5).

## 4 Discussion

The present study examined whether the *Magenstrasse*, a known shortcut for gastric emptying of liquids in the fed state, can influence absorption of a drug less soluble than caffeine. Under standardized postprandial conditions, we observed that caffeine (highly soluble) and theobromine (less soluble) appeared in saliva at nearly the same time after dosing, regardless of whether 50, 100, or 150 mL of water was co-administered. This finding indicates that theobromine, as a sparingly soluble compound compared to caffeine, is also emptied from the stomach via the *Magenstrasse*. Consistent with this, our MRI measurements confirmed that the ingested water emptied quickly (usually within ∼20 min) irrespective of the volume (Großmann et al., 2025). The fact that caffeine and theobromine had nearly concurrent onset in saliva suggests that the *Magenstrasse* pathway can entrain not only highly soluble drugs but also a sparingly soluble drug, possibly up to the extent that it is in solution. In practical terms, the stomach's postprandial liquid emptying route offers a means for immediate theobromine transit to the intestine despite the presence of food, supporting a rapid onset of plasma- or in our case saliva levels. Importantly, we found no significant influence of the initial water volume on overall drug exposure in this study. Even 50 mL of water were sufficient to trigger the *Magenstrasse* emptying, resulting in salivary theobromine profiles comparable to those with 100 mL or 150 mL. This aligns with recent evidence that the *Magenstrasse* occurs robustly even at smaller postprandial water volumes (Großmann et al., 2025). Taken together, these results highlight that a rapidly emptied liquid phase could facilitate early exposure for both compounds under fed conditions.

While theobromine displayed an initial absorption phase similar to caffeine, our data also revealed a distinct later phase of drug release and uptake for theobromine. After the first hour, when an additional water bolus was given, the salivary concentrations of theobromine rose substantially relative to caffeine. In the 59–119 min interval, theobromine's exposure exceeded caffeine's by a wide margin (post-hoc analysis showed ∼300 units greater dose-normalized AUC; *p* < 0.001). This divergent two-phase profile suggests that a significant portion of the drug remained in the stomach and dissolved gradually, only reaching the intestine and systemic circulation later when gastric emptying was forced with a second application of water. In contrast, the highly soluble caffeine was likely mostly dissolved, transported and absorbed during the early phase. Although theobromine cannot be clearly classified, these observations are in line with the known interplay between dissolution and gastric emptying for BCS II drugs. Literature reports have noted that if a dosage form requires substantial dissolution in the stomach, not all of the drug will empty with the initial liquid phase and undissolved fractions may sediment in the proximal stomach or become trapped in the viscous chyme, delaying their transit (Van Den Abeele et al., 2016; Danielak et al., 2024; Koenigsknecht et al., 2017; Dahan and Amidon, 2008; Koziolek et al., 2016). This mechanism could explain why the absorption of theobromine was prolonged despite the *Magenstrasse*-mediated initial emptying of water (Schick et al., 2020; Winter et al., 2024). The phenomenon aligns with prior studies showing that solids remain in the stomach during the initial postprandial period even as water empties rapidly (Koziolek et al., 2013; Vertzoni et al., 2019; Koziolek et al., 2019; Almutairi et al., 2023). However, future studies should extend the study protocol to be able to examine the effect of additional drinking after 60 min under persisting fasting conditions. Formal in vitro disintegration and dissolution testing was not performed in the present study. However, hard gelatin capsules were deliberately chosen over HPMC-based alternatives due to their well-established rapid aqueous disintegration, thereby minimizing formulation-related variability in content release (Jones et al., 2012; Sager et al., 2019). In vitro dissolution testing under postprandial conditions would moreover be of limited informative value, given that the complex physicochemical environment of the fed stomach, including meal composition, viscosity, and buffer capacity, differs substantially from standardized compendial media. Furthermore, formal particle size characterization of the encapsulated caffeine and theobromine batches was not performed. As particle size influences dissolution rate in accordance with the Noyes-Whitney equation, differences in particle morphology between the two compounds cannot be entirely excluded as a contributing factor to the observed differences in salivary appearance profiles.

It remains unclear to which extend particulate drug substances may also be evacuated by the *Magenstrasse*. If the dissolution rate of a lesser soluble drug substance is sufficiently high, an increased volume of co-administered fluid volume might increase the fraction of dose emptied via the *Magenstrasse*. Indeed, our results hinted that higher water volumes (100–150 mL) did not markedly boost the early exposure of theobromine relative to 50 mL, likely because even 50 mL was enough to initiate quick emptying and any dissolution advantage of extra fluid was marginal within the short timeframe and fast gastric water emptying. Due to the exponential emptying of water, it only takes a few minutes for different water volumes to reach the same intra-gastric volume, limiting the “extra”-time for dissolution under higher volumes of water (Großmann et al., 2025). The effect of 240 mL water on the dissolution and gastric emptying of theobromine was not studied. It is not possible to estimate the extend of this effect. In all three study arms, theobromine was not able to dissolve completely. This leaves most likely the slow dissolution rate being the reason that more water does not mean more dissolution. Due to insufficient mixing and the position of the dosage form in the stomach, the entire volume of applied liquid is not directly available for dissolution. However, a study with rats, has suggested that, for dosage forms with dissolution-limited drugs, increasing the co-administered water can be beneficial to prevent incomplete gastric transit of the dose (Kataoka et al., 2022). In our case 50 mL water might have been a cut-off set too high to find any influence of the applied water volume on early drug exposure. Although, using our described methods, an emptying of solid particles could not be shown here. However, this effect could explain parts of the uniform emptying and salivary appearance regardless of the fluid amount given. In practice, standard advice to take oral medications with a full glass of water is particularly appropriate for poorly soluble drugs, as it maximizes dissolution space and minimizes the risk of dosage form retention (Mudie et al., 2010; Alqahtani et al., 2021).

Our finding that theobromine showed slightly higher dose-normalized exposure than caffeine, despite its lower aqueous solubility, should be interpreted with caution, particularly as the difference was not statistically significant and the observations period was rather small. The most parsimonious explanation for the slightly higher dose-normalized AUC_0__–__119 min_ of theobromine is its lower systemic clearance relative to caffeine, resulting in a longer elimination half-life (∼7 h vs. ∼4 h) and consequently higher AUC/D values, independent of any dissolution or absorption differences. A classical positive food effect, as described for some BCS class II compounds, appears unlikely to be the main driver, given the already high oral bioavailability of theobromine and the limited scope for further absorption enhancement (FDA and CDER, 2002; Walton et al., 2001; Latini et al., 1984). Differences in distribution also do not provide a consistent explanation. Although theobromine exhibits a somewhat higher apparent volume of distribution, which would rather be expected to decrease normalized exposure, the observed trend points in the opposite direction (Rodopoulos et al., 1996; Walton et al., 2001; Arnaud, 1987; Zylber-Katz et al., 1984). Likewise, saliva-to-plasma partitioning (s/p ratio) is unlikely to account for the finding, as direct comparative data on saliva-to-plasma partitioning between theobromine and caffeine are lacking, and available evidence does not consistently indicate a substantially higher s/p ratio for theobromine. Metabolic interactions between both methylxanthines cannot be entirely excluded, particularly considering the formation of labelled metabolites from isotope-labelled caffeine (Walton et al., 2001). However, given the clear chromatographic separation of analytes prior to mass spectrometric detection, a relevant analytical bias due to signal overlap appears unlikely. Overall, the small numerical difference is most plausibly attributed to interindividual variability and the limited sample size rather than a distinct mechanistic effect. Minor contributions from postprandial dissolution dynamics cannot be ruled out, but are likely of secondary importance. In this context, the Magenstrasse may have facilitated an early absorption phase for both compounds by rapidly transporting dissolved fractions into the intestine, thereby reducing potential differences arising from solubility or gastric retention.

## 5 Conclusion

Gastric liquid emptying via the *Magenstrasse* occurs effectively under fed conditions even with as little as 50 mL of co-administered water. Both caffeine and theobromine showed rapid early salivary appearance independent of initial fluid volume, indicating that small water amounts are sufficient to trigger fast transit of dissolved drug fractions. However, theobromine exhibited a slightly slower increase in exposure after capsule opening and a pronounced late exposure increase after additional water intake, demonstrating that undissolved fractions may remain in the stomach and contribute to delayed absorption. Thus, while initial water volume did not critically influence early emptying in this setting, post-dose drinking clearly modified systemic exposure and contributed to biphasic absorption behavior of less-soluble theobromine. These findings furthermore suggest that standardization of post-dose drinking behavior should be considered in the design of food effect and bioavailability studies, as uncontrolled fluid intake after dosing may represent a relevant and underappreciated source of pharmacokinetic variability for sparingly soluble compounds.

## CRediT authorship contribution statement

**Linus Großmann:** Writing – original draft, Supervision, Project administration, Methodology, Investigation, Conceptualization. **Johanna Cyrus:** Writing – review & editing, Investigation. **Stefan Senekowitsch:** Writing – review & editing, Investigation. **Toni Wildgrube:** Writing – review & editing, Investigation. **Marie-Luise Kromrey:** Resources. **Werner Weitschies:** Writing – review & editing, Supervision, Conceptualization. **Philipp Schick:** Writing – review & editing. **Michael Grimm:** Writing – review & editing, Supervision, Conceptualization.

## Funding

The University of Greifswald received funding from the German Research Foundation (DFG, INST 292/155-1 FUGG).

## Declaration of competing interest

All authors declare that they have no known competing financial interests or personal relationships that could have appeared to influence the work in this publication.

## Data Availability

Data will be made available on request.
