# Supplementary material for: The effect of varying water volumes on in vivo dissolution and gastric emptying of highly soluble caffeine and lesser soluble theobromine containing capsules
Source: Int J Pharm X. 2026 Jul 15;12:100615. doi: 10.1016/j.ijpx.2026.100615 (PMC13400285; doi:10.1016/j.ijpx.2026.100615)
Supplement: Supplementary file 1 — Supplementary material [file mmc1.pdf]

Supplementary Material for:

The Effect of Varying Water Volumes on In Vivo Dissolution and Gastric Emptying of Highly Soluble Caffeine and Lesser Soluble Theobromine Containing Capsules

Linus Großmann[1], Johanna Cyrus[2], Stefan Senekowitsch[3], Toni Wildgrube[1], Marie-Luise Kromrey[4], Werner Weitschies[1], Philipp Schick[1], Michael Grimm[1]*

[1] University of Greifswald, Institute of Pharmacy, Dep. of Biopharmaceutics and Pharmaceutical Technology, Felix-Hausdorff-Str. 3, 17489 Greifswald, Germany

[2] Martin-Luther-University Halle-Wittenberg, Institute of Pharmacy, Dep. of Pharmaceutical Technology, 06099 Halle, Germany

[3] University Medicine Rostock, Institute of Pharmacology and Toxicology, Schillingallee 70, 18057 Rostock, Germany

[4] University Medicine Carl Gustav Carus, Institute of Diagnostic and Interventional Radiology, Fetscherstraße 74, 01307 Dresden, Germany

* Corresponding Author: michael.grimm@uni -greifswald.de

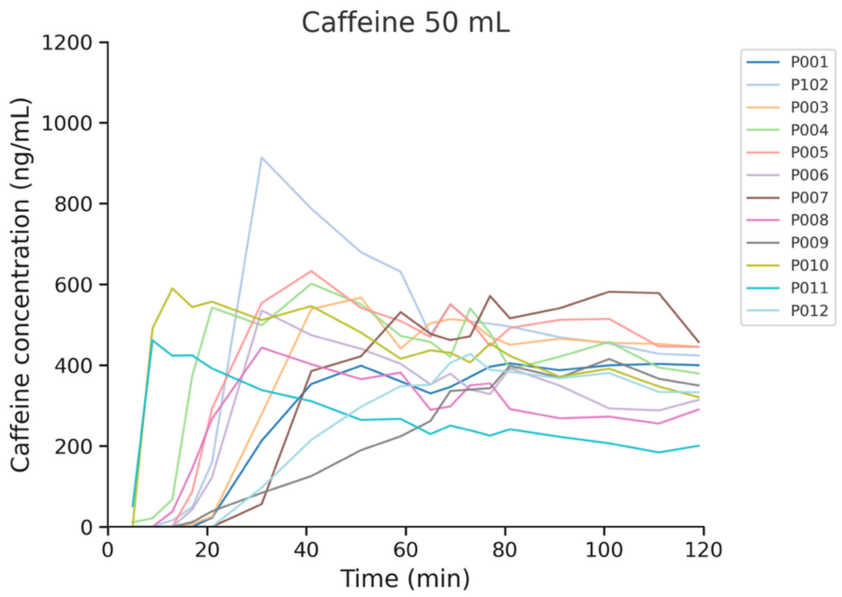

Figure S 1 Individual data of $^{13}C_3$-caffeine saliva concentrations after intake of 25 mg $^{13}C_3$-caffeine- and 50 mg theobromine-containing hard gelatine capsule with 50 mL water under fed conditions.

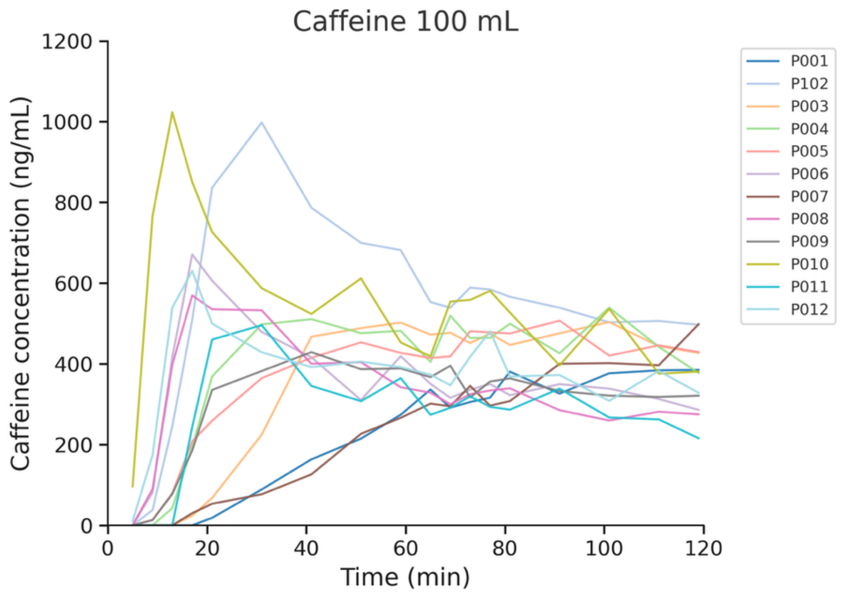

Figure S 2 Individual data of $^{13}C_3$-caffeine saliva concentrations after intake of 25 mg $^{13}C_3$-caffeine- and 50 mg theobromine-containing hard gelatine capsule with 100 mL water under fed conditions.

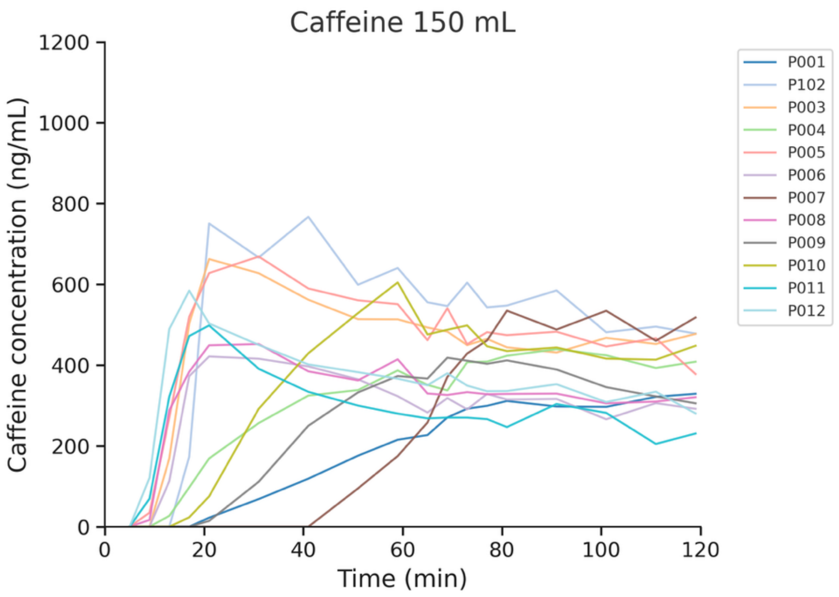

Figure S 3 Individual data of $^{13}C_3$-caffeine saliva concentrations after intake of 25 mg $^{13}C_3$-caffeine- and 50 mg theobromine-containing hard gelatine capsule with 150 mL water under fed conditions.

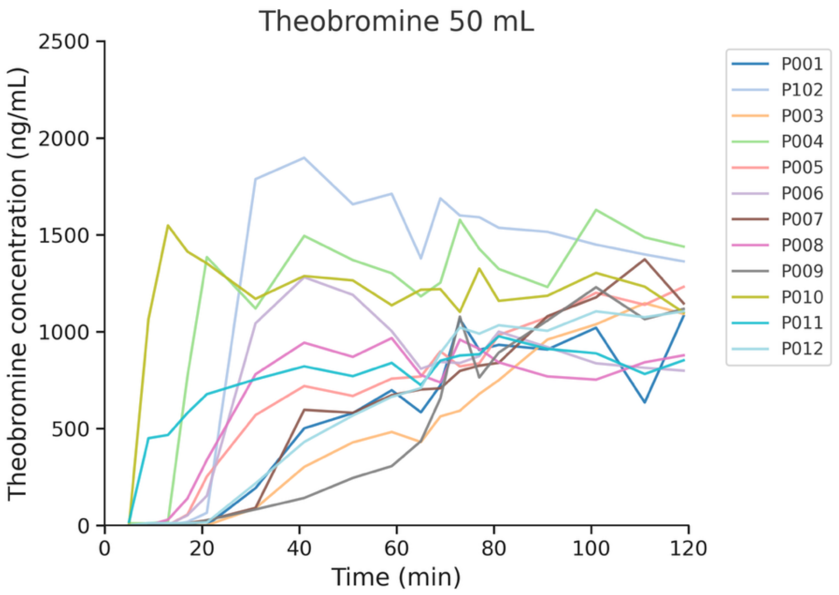

Figure S 4 Individual data of theobromine saliva concentrations after intake of 25 mg $^{13}C_3$-caffeine- and 50 mg theobromine-containing hard gelatine capsule with 50 mL water under fed conditions.

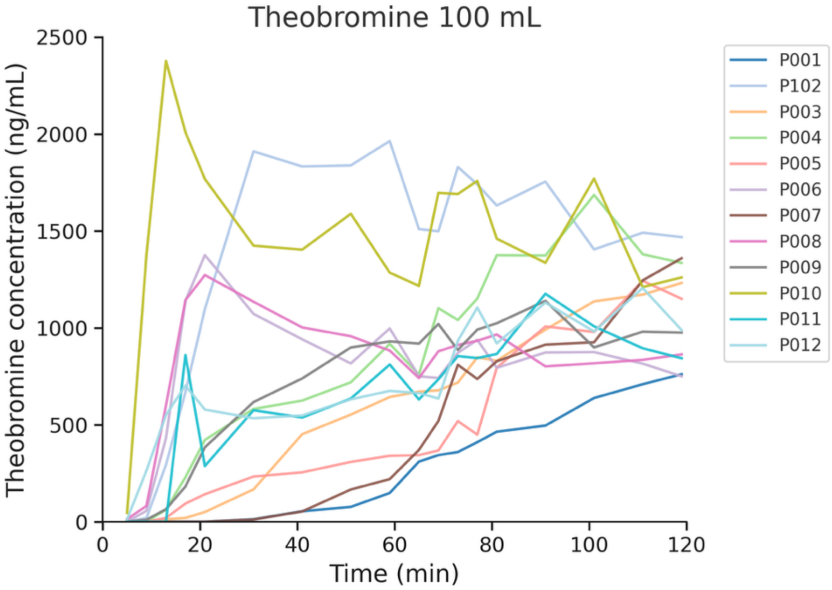

Figure S 5 Individual data of theobromine saliva concentrations after intake of 25 mg $^{13}C_3$-caffeine- and 50 mg theobromine-containing hard gelatine capsule with 100 mL water under fed conditions.

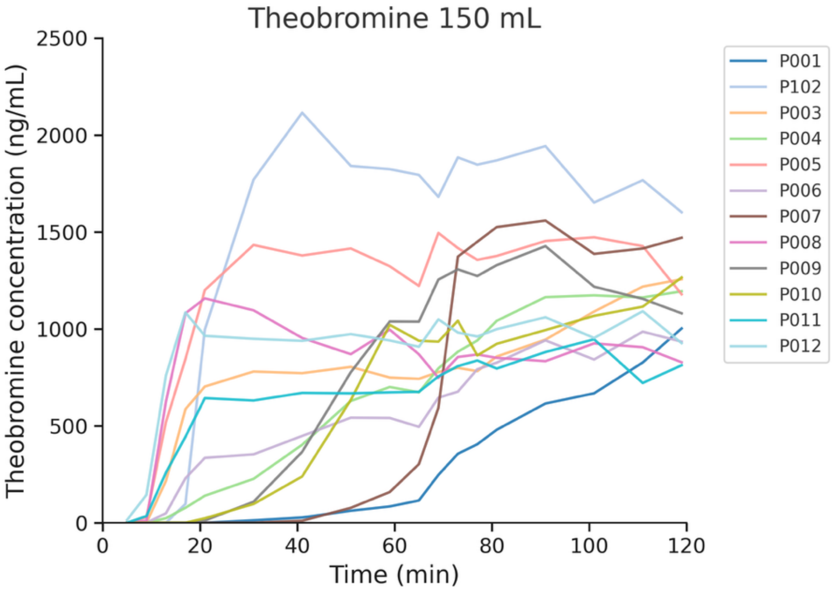

Figure S 6 Individual data of theobromine saliva concentrations after intake of 25 mg $^{13}C_3$-caffeine- and 50 mg theobromine-containing hard gelatine capsule with 150 mL water under fed conditions.

Table S 1 Bioanalytical validation results for the LC-MS/MS determination of theobromine in human saliva.

Validation parameter	Statistic	QC-LLOQ[1] (10 ng/mL)	QC-L[2] (25 ng/mL)	QC-M[3] (600 ng/mL)	QC-H[4] (1500 ng/mL)
**Within-run (n = 6)**	Accuracy (Mean ± SD, %)	115.33 ± 5.96	100.87 ± 3.79	93.15 ± 1.18	91.33 ± 2.45
	Precision (RSD, %)	5.17	3.76	1.27	2.69
**Between-run (n = 18)**	Accuracy (Mean ± SD, %)	109.99 ± 9.24	103.83 ± 7.26	94.32 ± 2.34	90.94 ± 5.03
	Precision (RSD, %)	8.40	6.99	2.49	5.53
**Freeze–thaw stability *(n = 4, 3 cycles)***	Accuracy (Mean ± SD, %)	–	96.37 ± 5.21	–	92.72 ± 2.42
	Precision (RSD, %)	–	5.41	–	2.61
**Rack stability**	Accuracy (Mean ± SD, %)	107.55 ± 7.93	101.43 ± 6.59	97.97 ± 0.88	95.25 ± 1.58
	Precision (RSD, %)	7.37	6.50	0.90	1.66
**Bench-top stability (24 h)**	Accuracy (Mean ± SD, %)	–	105.80 ± 7.23	–	96.18 ± 4.07
	Precision (RSD, %)	–	6.84	–	4.23

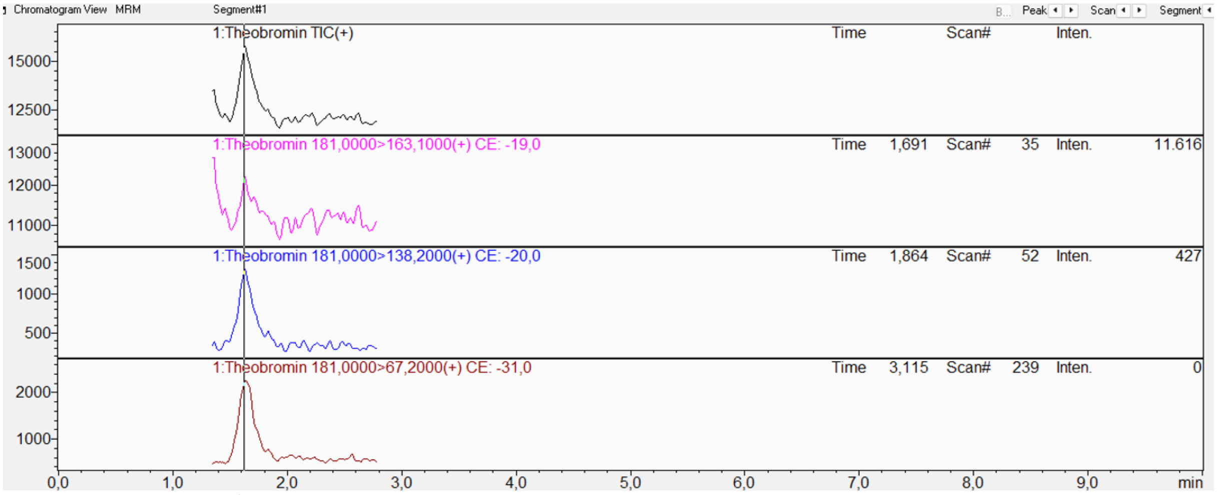

Figure S 7 [1]*QC-LLOQ (S/N 7.09)*

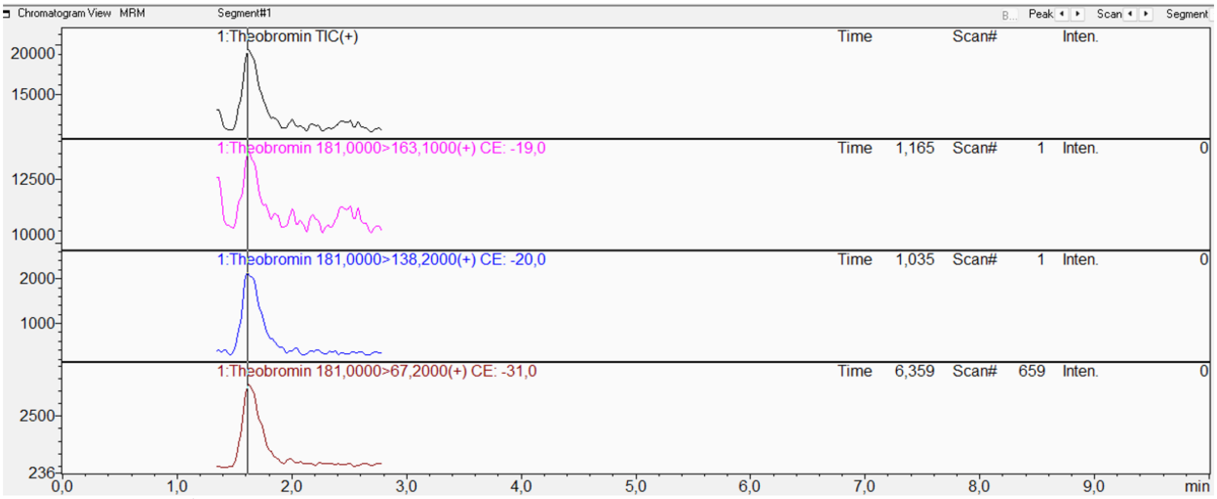

Figure S 8 [2]QC-L (S/N 17.30)

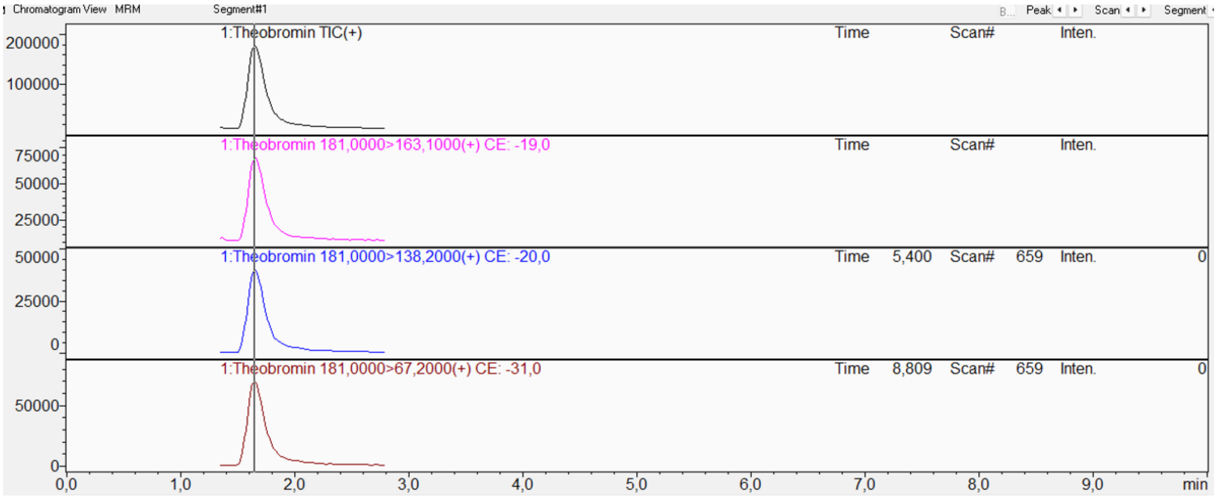

Figure S 9 [3]*QC-M (S/N 116.98)*

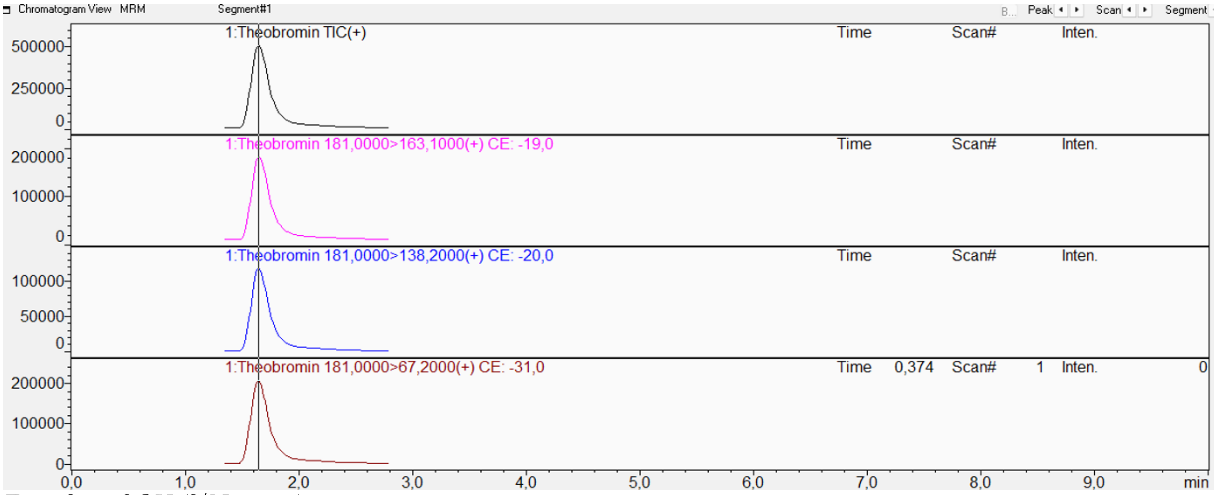

Figure S 10 4QC-H (S/N 262.41)
